# MN1–Fli1 oncofusion transforms murine hematopoietic progenitor cells into acute megakaryoblastic leukemia cells

**DOI:** 10.1038/oncsis.2015.41

**Published:** 2015-12-21

**Authors:** D V Wenge, E Felipe-Fumero, L Angenendt, C Schliemann, E Schmidt, L H Schmidt, C Thiede, G Ehninger, W E Berdel, M-F Arteaga, J-H Mikesch

**Affiliations:** 1 Department of Medicine A, Hematology and Oncology, University of Münster, Münster, Germany; 2 Department of Medicine I, University of Dresden, Dresden, Germany

## Abstract

Long-term outcome of acute megakaryoblastic leukemia (AMKL) patients without Down's syndrome remains poor. Founding mutations and chimeric oncogenes characterize various AMKL subtypes. However, for around one third of all cases the underlying mechanisms of AMKL leukemogenesis are still largely unknown. Recently, an in-frame fusion of meningeoma 1–friend leukemia virus integration 1 (*MN1*–*Fli1*) gene was detected in a child with AMKL. We intended to investigate the potential role of this oncofusion in leukemogenesis of acute myeloid leukemia. Strikingly, expression of MN1–Fli1 in murine hematopoietic progenitor cells was sufficient to induce leukemic transformation generating immature myeloid cells with cytomorphology and expression of surface markers typical for AMKL. Systematic structure function analyses revealed FLS and 3′ETS domains of *Fli1* as decisive domains for the AMKL phenotype. Our data highlight an important role of MN1–Fli1 in AMKL leukemogenesis and provide a basis for research assessing the value of this oncofusion as a future diagnostic marker and/or therapeutic target in AMKL patients.

## Introduction

Acute megakaryoblastic leukemia accounts for up to 2% of acute myeloid leukemia (AML) in adults. Distinct AMKL subtypes are characterized by founding mutations and chimeric oncogenes.^1, 2, 3^ Prognosis of pediatric AMKL patients with Down's syndrome (DS) is favorable. However, very little is known about leukemogenesis of non-DS AMKL, and the outcome of these patients remains poor. Transcriptome sequencing of AML cells from pediatric AMKL patients revealed that AMKL is characterized by chimeric transcripts.^2^ Various fusion proteins have been described in AMKL.^2, 4, 5^ Recently, in one case of pediatric AMKL a novel in-frame fusion of meningeoma 1–friend leukemia virus integration 1 (*MN1*–*Fli1*) gene was identified.^2^ In adult AML overexpression of both, *MN1* and *Fli1* is frequently found and confers a poor prognosis to the patients.^6, 7, 8^ Moreover, Fli1 is a known key transcription factor in megakaryopoiesis.^9^ Thus, we intended to investigate the role of MN1–Fli1 in adult AMKL development.

## Results and discussion

**Figure 1:**
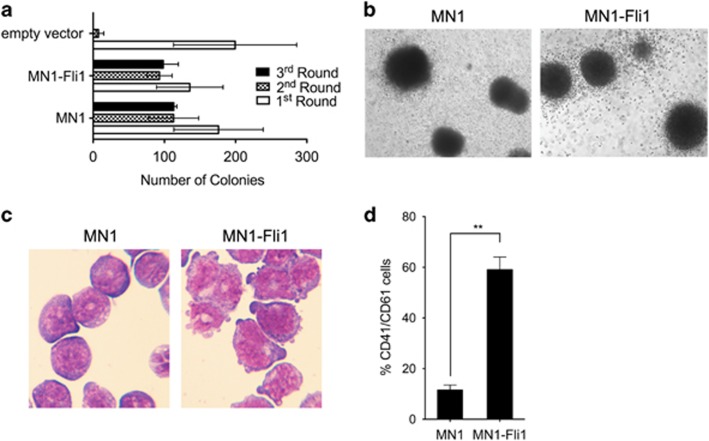
MN1–Fli1 induces transformation of murine HPCs generating myeloid leukemia cells with AMKL phenotype. (**a**) Bar charts represent first- to third-round colony numbers as assessed by retroviral transduction and transformation assays. In brief, c-kit^+^ hematopoietic progenitor cells (HPCs) were cultured overnight in RPMI 1640 medium, 10% fetal calf serum (FCS) and 2 mM l-glutamine, supplemented with 20 ng/ml stem cell factor (SCF), 10 ng/ml IL-3 and IL-6. Spinoculation was carried out by centrifugation at 800 *g* (32 °C, 2 h) with 5 μg/ml polybrene (Sigma-Aldrich, Saint Louis, MO, USA). Cells were plated in M3234 methylcellulose medium (Stem Cell Technologies, Vancouver, BC, Canada) supplemented with recombinant murine 20 ng/ml SCF, 10 ng/ml IL-3, IL-6 and granulocyte macrophage colony-stimulating factor (GM-CSF) (PeproTech EC, London, UK) and antibiotic on the following day. Colonies were scored and replated weekly. Retroviral plasmids were generated by direct cloning or PCR-based site-directed mutagenesis and verified by DNA sequencing. Error bars represent s.d. from at least three independent experiments. (**b**) Typical morphology of third-round colonies from primary murine bone marrow (BM) cells transduced with MN1 or MN1–Fli1. (**c**) May–Giemsa staining of cytospins of primary BM cells transformed with MN1 or MN1–Fli1. (**d**) Immunophenotype analysis of primary BM cells transformed by MN1 or MN1–Fli1. Fluorochrome-conjugated monoclonal phycoerythrin (PE) or fluorescein isothiocyanate (FITC) conjugated antibodies to murine CD41 (clone MWReg30) and CD61 (clone 2C9.G2) (Pharmingen BD Biosciences, San Diego, CA, USA) were used. Protocols and reagents used were as previously described.^14^ Histographs represent staining obtained with antibodies specific for murine CD41 and CD61. Error bars represent s.d. from at least three independent experiments. Two-tailed Student's *t*-test was used to determine statistical significance (**P<0.05).

**Figure 2:**
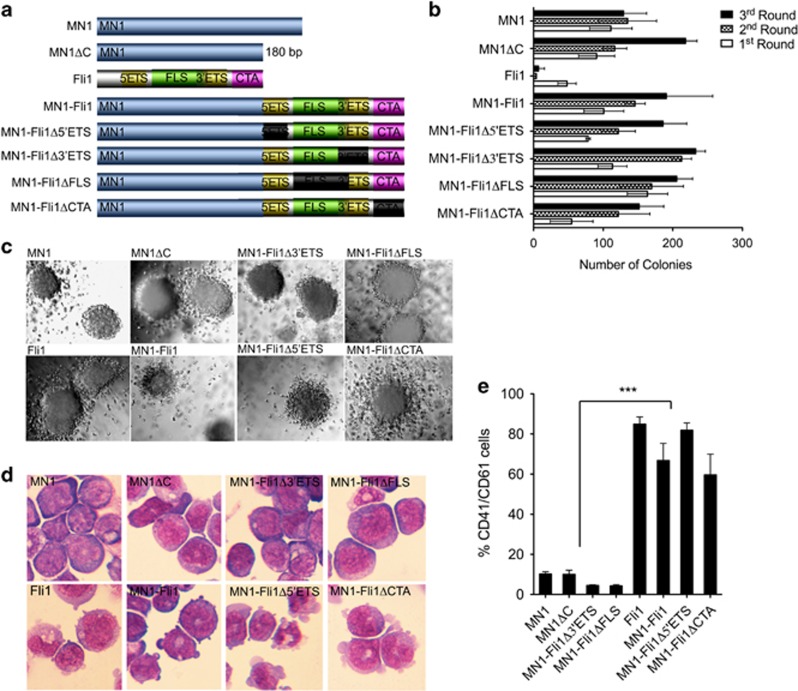
FLS and 3′ETS domains of Fli1-mediate AMKL phenotype to MN1–Fli1 associated leukemic transformation. (**a**) Schematic diagram shows MN1, Fli1 and according mutants used for retroviral transduction and transformation assays (RTTA). (**b**) Bar charts represent first to third round colony numbers generated via RTTA with the indicated constructs. Error bars represent s.d. from at least three independent experiments. (**c**) Typical morphology of third round colonies from primary bone marrow (BM) cells. (**d**) May–Giemsa staining of cytospins of cells derived from third round colonies of primary BM cells transformed with the indicated constructs. (**e**) Immunophenotype analysis of cells transformed by the indicated constructs. Histographs represent staining performed with antibodies specific for murine CD41 and CD61. Error bars represent s.d. from at least three independent experiments. One-way analysis of variance (ANOVA) was used to determine statistical significance (***P<0.0001).

To determine the role of MN1–Fli1 in AMKL, we retrovirally expressed MN1, MN1–Fli1 or an empty vector control in primary murine c-kit^+^ hematopoietic progenitor cells (HPCs) derived from the bone marrow of C57Bl/6 mice and assessed their effect on *in vitro* colony replating capacity as a surrogate measure of self-renewal. Expression levels of *MN1* and *MN1-Fli1*, respectively, were similar as confirmed via quantitative reverse transcription–PCR (Supplementary Figure 1). In contrast to cells transduced with vector control, those transformed by MN1 could be serially replated to form compact colonies beyond the third round of replating (Figures 1a and b; and data not shown) generating myeloid leukemia cells as shown previously.^10^ Interestingly, MN1–Fli1-transduced cells were able to form similar numbers of compact colonies resembling to MN1-derived colonies beyond the third round of replating (Figures 1a and b; and data not shown). However, May–Giemsa staining of cytospins of MN1 and MN1–Fli1 third round cells revealed strong differences in terms of morphology and expression of cell surface markers. Although MN1-transduced cells displayed their known myeloid blast morphology (Figure 1c), MN1–Fli1-transformed cells showed the phenotype of immature megakarypoietic cells, and mostly megakaryoblasts (Figure 1c). Moreover, flow cytometry analysis of these cells showed significant differences in terms of cell surface marker expression. The large majority of MN1–Fli1-transduced cells was positive for both CD41 and CD61, two characteristic AMKL markers, whereas double positivity for these markers was largely absent in MN1-transduced cells (Figure 1d). As a next step, we performed structure function analyses to identify potential domains mediating AMKL phenotype to HPCs transformed by MN1–Fli1. The MN1–Fli1 oncoprotein is the in-frame fusion product of N-terminal MN1 sequence, a transcriptional coactivator, fused to C-terminal Fli1 sequence, an E26-transformation-specific (ETS) transcription factor. MN1–Fli1 contains almost the entire *MN1* coding sequence at the N-terminal part and >70% of the *Fli1* gene at the C terminus.^2^ In total, MN1–Fli1 misses 180 bp of the MN1 C-terminus due to chromosomal breakage on chromosomes 22 (MN1) and 11 (Fli1) with subsequent gene fusion (Figure 2a). The *Fli1* gene harbors four distinct functional domains including the 5′ETS domain, a Fli-1-specific region (FLS), a 3′ETS domain responsible for sequence-specific DNA-binding activity and a carboxy-terminal transcriptional activation (CTA) domain involved in transcriptional activation and protein–protein interaction (Figure 2a).

To assess which parts of the MN1 or Fli1-coding sequence are connected with the AMKL phenotype in MN1–Fli1-transduced HPCs, we generated five different MN1 and MN1–Fli1 deletion mutants respectively: MN1ΔC (missing the 180 bp at the MN1 C-terminus also missing in MN1–Fli1), as well as MN1–Fli1Δ5′ETS, MN1–Fli1ΔFLS, MN1–Fli1Δ3′ETS and MN1–Fli1ΔCTA (Figure 2a). Again, similar expression levels of MN1, MN1–Fli1 and their mutants were confirmed via quantitative reverse transcription–PCR (Supplementary Figure 1). As a next step, we compared the transformation ability of these mutants with wt MN1 and wt Fli1 in primary murine HPCs via serial replating. As expected, all variants of MN1 and MN1–Fli1 could efficiently transform and enhance the replating ability of the HPCs as determined via retroviral transduction and transformation assays. All MN1, Fli1 and MN1–Fli1 could form colonies with similar morphology in the third and subsequent rounds of replating (Figures 2b and c; and data not shown). Apart from wt Fli1-transduced cells showing reduced third round colony numbers as compared with all other MN1 and MN1–Fli1 variants, transformation capacity of all constructs was comparable (Figure 2b). Strikingly, May–Giemsa staining and flow cytometry revealed that both, FLS and 3′ETS domain of Fli1 determined the AMKL phenotype of MN1–Fli1-transduced murine HPCs. Whereas, AMKL cytomorphology as well as high expression of CD41/CD61 were both maintained in MN1–Fli1Δ5′ETS and MN1–Fli1ΔCTA-transduced cells, morphology of those HPCs transduced with MN1–Fli1ΔFLS or MN1–Fli1Δ3′ETS strongly resembled to MN1 and MN1ΔC-transduced cells. Moreover, MN1–Fli1ΔFLS and MN1–Fli1Δ3′ETS cells significantly lost expression of the two typical AMKL surface markers (Figures 2d and e).

Screening peripheral blood and bone marrow samples of 33 adult AMKL patients via PCR, we could not detect any patient with expression of MN1–Fli1 (data not shown). Thus, to finally determine the frequency and age distribution of this oncofusion in AMKL, analysis of a larger patient cohort including pediatric patients will be required.

The *Fli1* gene encodes a member of the ETS family of transcription factors, which have an important role in hematopoiesis including the regulation of hematopoietic stem/progenitor cell maintenance and differentiation.^11, 12^ Moreover, aberrant expression of Fli1 is involved in myeloid leukemias and various types of human cancers including Ewing sarcoma with balanced chromosomal translocations generating the EWS-Fli1 oncofusion.^12^ These studies implicate Fli1 and its signaling pathways as candidates for future targeted cancer therapies.^12, 13^ Fli1 binds to DNA in a sequence-specific way. Both, the FLS domain as well as the 3′ETS domain of Fli1 contribute to DNA binding suggesting that MN1–Fli1-mediated leukemic transformation generates an AMKL phenotype via abnormal transcriptional activation of Fli1. We speculate that Fli1 fused to MN1 is redirected toward specific target gene promoters via FLS and 3′ETS domain dependent DNA binding determining the AMKL phenotype.

Our study underscores a potential key role for the MN1–Fli1 oncofusion in AMKL leukemogenesis. Future studies may determine a potential diagnostic and/or prognostic role of MN1–Fli1 expression in AML as well as investigate its value as a putative marker for remission control in a subgroup of AMKL patients. Further investigation is needed to identify the major target genes of MN1–Fli1. Targeting either Fli1 directly or its key target genes may enable development of future therapeutic strategies for non-DS AMKL patients representing the poor prognosis subgroup of AMKL.

## Abbreviations

AMKL: Acute megakaryoblastic leukemia
AML: Acute myeloid leukemia
CTA: Carboxy-terminal transcriptional activation
DS: Down's syndrome
ETS: E26 transformation-specific
Fli1: Friend leukemia virus integration 1
FLS: Fli-1-specific region
HPC: hematopoietic progenitor cell
MN1: Meningeoma 1.
