# Supplementary material for: MN1–Fli1 oncofusion transforms murine hematopoietic progenitor cells into acute megakaryoblastic leukemia cells
Source: Oncogenesis. 2015 Dec 21;4(12):e179–. doi: 10.1038/oncsis.2015.41 (PMC4688398; doi:10.1038/oncsis.2015.41)
Supplement: Supplementary Figure 1 [file oncsis201541x1.doc]

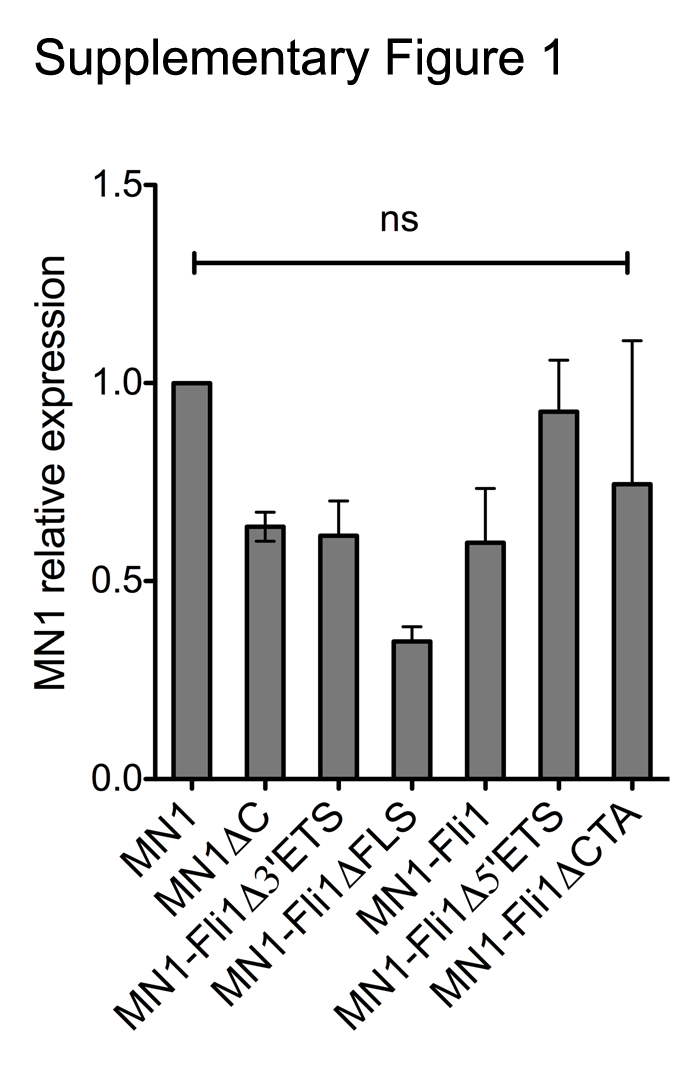


**Supplementary Figure 1:**

qRT-PCR for MN1 expression in primary murine bone marrow cells transduced with the indicated constructs. Sequences of the primers used for qPCR amplification: hMN1for: attgacctggactcgctgatg, hMN1rev: tgctgaggccttgtttgca. mGapdh expression was used for normalization. Error bars indicate SD of at least three independent experiments. Statistical significance was assessed by one-way ANOVA with Prism GraphPad software (ns: not significant).
